# An AI-powered Electronic Nose System with Fingerprint Extraction for Aroma Recognition of Coffee Beans

**DOI:** 10.3390/mi13081313

**Published:** 2022-08-13

**Authors:** Chung-Hong Lee, I-Te Chen, Hsin-Chang Yang, Yenming J. Chen

**Affiliations:** 1 Department of Electrical Engineering, National Kaohsiung University of Science and Technology, Kaohsiung 807618, Taiwan; 2 Department of Healthcare Administration and Medical Informatics, Kaohsiung Medical University, Kaohsiung 80708, Taiwan; 3 Department of Medical Research, Kaohsiung Medical University Chung-Ho Memorial Hospital, Kaohsiung 80756, Taiwan; 4 Department of Information Management, National University of Kaohsiung, Kaohsiung 811726, Taiwan; 5 Department of Information Management, National Kaohsiung University of Science and Technology, Kaohsiung 824005, Taiwan

**Keywords:** electronic nose, sensor fusion, coffee aroma, machine learning

## Abstract

Aroma and taste have long been considered important indicators of quality coffee. Specialty coffee, that is, coffee from a single estate, farm, or village in a coffee-growing region, in particular, has a unique aroma that reflects the coffee-producing region. In order to enable the traceability of coffee origin, in this study we have developed an e-nose system to discriminate the aroma of freshly roasted coffee in different production regions. In the case study, we employed the e-nose system to experiment with various machine learning models for recognizing several collected coffee beans such as coffees from Yirgacheffe and Kona. Additionally, our contribution also includes the development of a method to create an aromatic digital fingerprint of a specific coffee bean to identify its origin. The experimental results show that the developed e-nose system achieves good recognition performance for coffee aroma recognition. The extracted digital fingerprints have great potential to be stored in an extensible coffee aroma database similar to a comprehensive library of specific coffee bean aroma characteristics, for traceability and reconfirmation of their origin.

## 1. Introduction

Drinking coffee is a part of daily life for many people. The aroma and taste of coffee are mainly produced after high-temperature roasting. During the roasting process, the moisture of the green coffee beans is slowly released and a large amount of the chlorogenic acid originally contained in green coffee beans will gradually disappear during the process of high-temperature roasting. The taste of coffee also varies with the length of time it has been roasted. More recently, the electronic nose (e-nose) [1] has been used as an alternative to traditional methods for evaluating aromas or other chemometrics in food and beverages such as wine [2,3], meat [4], juices [5,6], and tea [7,8,9]. The e-nose was originally designed and proposed in the early 1980s by Persaud [1], who developed an e-nose using semiconductor sensors and found that it was able to distinguish between various odors. In current e-nose systems, sensor array methods and machine learning algorithms have been used to classify odors [10]. The sensory properties of coffee are deeply influenced by the composition of the coffee beans, mainly due to the climatic characteristics associated with different latitudes and altitudes. As coffee is grown and produced around the world, the characteristics of coffee beans have become more unique from one geographic location to another. Nuances in soil, microclimate, and environmental factors give coffee beans their unique characteristics, affecting their flavor, body, aroma, and acidity. However, such variability may also increase the risk of fraudulent practices such as mislabeling products to hide the true geographic origin of the coffee beans. Therefore, it is necessary to develop analytical methods that can effectively assess the geographic origin of coffee beans. This work combines techniques from olfactory sensor arrays and machine learning techniques to identify the aromas of freshly roasted single-origin coffees from different origins.

The specific aroma of coffee is determined by the variety and the high-temperature roasting process. The roasting process variables are set one by one to improve the reproducibility of the quality of single-origin coffees. During roasting, the green coffee beans produce a large amount of carbon dioxide and moisture from heating which starts the caramel chemical reaction (the oxidation and browning reaction of sugar) and the Mena reaction (the browning reaction of amino acids and carbohydrates). These are key to the aroma that is generated. To maintain the quality of the roasted coffee beans, it is first necessary to trace the origin of the green beans because it is difficult to maintain a consistent quality of green coffee beans. Even the sunshine, rainfall, temperature, and related conditions of the same farm cannot be consistent every year, which indirectly affects the aromatic ingredients of the harvested green coffee beans. In addition, the temperature, humidity, and weather conditions during the roasting process affect the aroma. In order to obtain accurate data during the collection of gas data for the odor analysis, the system must be isolated from the ambient air. However, the roasted coffee beans need to emit carbon dioxide to release aromas to make the flavor more pronounced, and the data preprocessing and classifier type also affect the gas classification and identification results. In the past, the data preprocesses the specific signal data from the gas response signals as features and uses machine learning algorithms such as k-nearest neighbor (K-NN) [11] or support vector machine [12] to classify the gas data. In recent years, there has been literature describing the use of deep learning for gas classification [13]. In data preprocessing, the process of the gas reaction has been used as a feature and the convolutional neural network method has been used to classify the gases.

The motivation behind this work is to prevent the incidents where unscrupulous merchants mislabel inferior coffee beans as high-end beans to deceive consumers. Due to their different origins, coffee producers usually classify their own products, especially with regard to the main aromas that affect coffee quality; this process ensures that consumers associate the specific aroma of coffee with its origin, which is beneficial for increasing market sales. Therefore, it is useful to develop a method to easily verify the origin of coffee beans to ensure quality. In this work, we utilized our developed electronic nose (e-nose) system to collect aroma sensory data and then used several machine learning methods for the recognition task. In particular, the method allows us to cumulatively extract coffee aroma fingerprints in order to build a scalable database containing a wider variety of coffee bean aromas, thereby improving the recognition accuracy of the system.

The main contribution and goal of this work can be divided into the following:

- To use the extracted aroma data of coffee beans and machine learning techniques to ensure the authenticity of the origin of coffee beans.
- To establish a method for creating individual coffee aroma signatures (i.e., digital fingerprints) to identify their provenance.

In this study, we utilized the above-mentioned approaches to create an e-nose system for the aroma recognition of freshly roasted coffee beans. The data processing method involves converting the signals detected by each gas sensor into an aroma dataset but it lacks aroma digital fingerprints. Our algorithms measure and quantitatively represent the raw data in the form of digital fingerprints. These digital fingerprints have the potential to be stored in an extensive coffee aroma database, similar to a comprehensive library of specific coffee bean aroma characteristics, for the traceability and reconfirmation of their origin.

The rest of the paper is organized as follows. Section 2 surveys several related works in the literature. The proposed system framework, experimental process, and configuration of the e-nose system are described in Section 3. Section 4 reports on the feature extraction of aroma data for digital fingerprint generation. In particular, we introduce our developed method for the construction of the digital fingerprints of the measured coffee aromas. In Section 5, we describe our experiments and evaluate the performance of the method for the recognition of different coffee bean aromas using various machine learning models. In addition, the experimental results of digitizing coffee aromas with the proposed fingerprint generation technique are presented. Furthermore, the discussion of the experimental results is addressed in detail. Section 6 concludes the work by summarizing our research contributions.

## 2. Related Work

Several data fusion and machine learning algorithms for identifying aromas in coffee, beverages, and food have been discussed in the literature ranging from basic statistical techniques to sophisticated artificial-intelligence-based approaches. In particular, the emerging artificial intelligence method of deep learning has a better capability for learning hierarchical features than previous machine learning models. Deep-learning-based models provide an end-to-end learning framework to jointly learn feature representations while performing classification or recognition tasks. The use of deep learning (DL) has been applied to different industries such as deep learning in neural networks [14,15], medical image semantic segmentation based on deep learning [16,17], and deep-learning-based emotion analysis of microblog texts [18,19,20]. These studies are discussed below.

Wakhid [21] used several sensors combined with machine learning algorithms to accurately detect the odor variations in civet and other types of coffee beans and obtained a high accuracy of 97% in the final classification task using decision tree algorithms. The work of Harsono [22] studied musk coffee, a kind of rare and expensive coffee with a special flavor after digestion by animals. In order to confirm adulteration and quantify coffee beans, the Logister (LR) and Linear Discriminant Analysis (LDA), and K-NN methods were used, respectively. According to the analysis of the average value, standard deviation, and maximum value of the statistics obtained and the five olfactory sensors used to identify the mixed beans of Arabica civet coffee and Robusta coffee, the best classification was performed using the K-NN with an accuracy of 97.7%. Matteo Pardo [23] used Principal Component Analysis (PCA) and Multilayer Perceptron (MLP) to analyze seven types of single and mixed coffee beans and classify them according to the aroma after grinding and the accuracy rates were all above 90%; the average was 97.16%. Magfira [24] studied two of the world’s three major native coffee species, Arabica and Robusta. The third major species, Liberica, was not included in the study due to the fact that it was difficult to obtain information about its production. Only one olfactory sensor and SVM were used to classify the aroma of Arabica and Robusta coffee beans and the resulting accuracy was 71%. Omatu [25] used Learning Vector Quantization (LVQ) and 14 olfactory sensors in tea classification with an accuracy of 96% and coffee classification with an accuracy of 89%. Thazin [26] classified the acidity of freshly roasted coffee beans using a “cup test”. In the study, eight olfactory sensors were used to classify coffee acidity. Through PCA analysis, a coffee acidity classification was performed and Radial Basis Function (RBF) Neural Network was used to predict coffee acidity and the results show 95% accuracy. Tisan [27] proposed a classification module to implement a neural network on a Field Programmable Gate Array (FPGA) using seven olfactory sensors to identify four different types of released organic compounds from coffee and the recognition rate reached 94.57%. Rodríguez [28] used cupping analysis to detect and classify defects in Colombian coffee. The results obtained by the author showed that e-nose technology could be used to evaluate the quality of Colombian coffee for quality control, followed by PCA and neural networks to process the data. Macasaet [29] used 10 gas sensors to form an e-nose in order to simulate the olfactory system. This system can classify 10 kinds of odors and use artificial neural networks to identify and classify them. The results showed that four odors were identified and that the accuracy rates obtained using F-measure were spicy, 68.24%; chemical, 87.77%; mint, 54.32%; and rot:], 87.78%. Brudzewski [30] used an e-nose to identify coffee applications. The e-nose used in the experiment was a differential e-nose, which can capture signals from 11 different mixtures of Arabica and Robusta coffee and then process the signal data. The data set is randomly divided into 65% training data and 35% test data. A support vector machine (SVM) method was used to classify 12 signal data, and the average error of the results reached 0.21%. A study by Okur [31] consisted of three quartz crystal microbalance (QCM) sensor arrays that comprised the e-nose used in the research. These sensors are coated with surface-mounted metal–organic framework (SURMOF) film and use machine learning K-NN technology for data processing. Predictive analysis showed that the accuracy rates for the different pure odors and their mixtures reached 99.97% and 98.4%, respectively. The experimental results showed that the QCM sensor coated with SURMOF film could identify the odor of vegetable oil and its mixtures. In order to ensure good food quality and health benefits, Kukade [32] used eight olfactory sensors to identify cinnamon, cloves, and nutmeg using the Random Forest algorithm, with an accuracy rate as high as 100%, and the accuracy rate was also as high as 100% using MLP recognition. Liu [33] applied a portable e-nose with 10 sensors to 20 wines with 8 different flavors and the accuracy of the SVM method on the test data set reached 83.3%. The combination of appropriate sensors and machine learning models could additionally be used for the objective evaluation of liquor so an e-nose could potentially be used as a tool for objectively evaluating the quality control and flavor of Chinese liquor. Li [34] proposed a simple method to classify liquor, beer, coffee, and tea, using six olfactory sensors and imported the resistance change data into an Excel table. Yu [35] proposed a Back Propagation Neural Network (BPNN) method and 6 olfactory sensors to identify 14 different wine regions with an accuracy of 94.5%. Wu [36] used 16 olfactory sensors and a CNN model to predict odors and used 2 convolutional layers and 1 fully connected layer to predict odor pleasure. The performance was very good with an accuracy of 99%. Tang [37] developed a portable e-nose prototype consisting of eight commercial sensors, a data acquisition interface, and a microprocessor. In order for the system to recognize the aroma of different fruit, the K-NN algorithm was used to analyze the aroma of lemons, bananas, and lychees. The verified experimental results reached a classification accuracy of more than 95%. Sanchez-Lengeling [38] used Graph Neural Networks (GNN) to predict molecular properties. They employed GNN to predict the description of 138 odor molecules and the classification accuracy for AUROC was as high as 0.894 (89.4%). Wijaya [39] used an e-nose to obtain signal data combined with Discrete Wavelet Transform and Long Short-Term Memory (DWT–LSTM) to perform quality monitoring and category classification of 12 different beef data sets. The obtained average accuracy and F-measure were 94.83% and 85.05%. Qi [40] used the Deep Neural Network (DNN) model and seven olfactory sensors to identify 4 odors (tasteless, beer, whiskey, and red wine) in the study, with an average accuracy of 99.26%. Jong [41] used deep learning to identify the aroma patterns of four alcoholic beverages in the study and converted the data from nine olfactory sensors into standard deviation images. Using the GoogLeNet and AlexNet models, its recognition accuracy was 85.0%. Shi [42] proposed a combination of Convolutional Neural Network (CNN) and SVM methods to obtain data using e-nose sensors and then obtain beer signals from deep learning feature extraction. The olfactory features of beer were extracted through the convolutional layer and pooling layer in the CNN, whereas the SVM replaced the fully connected layer to enhance the model. The experimental results showed that the CNN–SVM model could automatically extract the olfactory characteristics of beer and achieved an accuracy of 96.67% in the test set. Müller [43] used ion mobility spectroscopy (IMS)-based e-nose measurements to classify odors. In order to avoid the time-consuming task of updating the database, the K-NN classifier in machine learning was used and PCA was added to accelerate the classification. Based on the IMS e-nose, the classification accuracy of the K-NN model was improved. The results showed that the use of K-NN did not significantly improve the classification accuracy but it reduced the classification time and the use of PCA sped up the classification process. Ma [44] proposed a model based on the dynamic response matrix of the sensor array, which obtained a smaller regression error. In their experiment, the VGG-19 model was used to identify different gases or Volatile Organic Compounds (VOC). The experimental results showed that with the sensor array of the e-nose, the model reached an accuracy of 90% and the accuracy of the correct normalization method increased to 94%. Liu [45] used 10 olfactory sensors to classify 6 levels of organic green tea. Using support vector machines and Random Forest classification models, the classification accuracies were 100% and 99.33%, respectively. Bhattacharyya [46] conducted electronic nasal experiments in different seasons and locations in India and mapped the PCA electronic detection range according to the time of use. An e-nose was used to monitor the integral emission mode of the black tea fermentation process and obtain the best fermentation time according to the output of the sensor. Due to its very high integrity and timeliness, an e-nose is more suitable for black tea processing. Dutta [8] used an e-nose based on a metal oxide sensor (MOS) to analyze five different qualities of tea. The technology used four MOS sensors, each of which is sensitive to tea samples. After the signal obtained by the sensor array was composed of tea sample data, the data was analyzed using neural networks such as RBF, PNN, and MLP. The results showed that RBF could accurately classify tea samples of five different qualities. Wang [47] designed an optimized Deep Convolutional Neural Network (DCNN) for the characteristics of e-nose data. This model optimized the convolutional layer and the pooling layer. In this study, 10 different Chinese herbal medicines of Dendrobium were identified, and the sensed data were extracted using an e-nose and then classified using the DCNN model. The experimental results showed that the optimized DCNN achieved the highest average accuracy of 87.56%. Zhang [48] employed e-nose data processing to simplify the process and improve performance and proposed a data processing method based on a sensor for the non-specific analysis of the data characteristics of liquor, and then used a channel-based attention Channel Attention Convolutional Neural Network (CA-CNN) for the identification. The experimental results showed that CA-CNN had better performance in liquor quality classification and the obtained liquor classification accuracy rate reached 98.53%. Wei [49] used a MOS-based e-nose to detect the palm core of Chinese pears and then used BP-Extreme Learning Machine Neural Network (BP-ELMNN) and PCA to extract the principal component features. The experimental results showed that the accuracy and F1-score obtained using this method were 96.83% and 96.85%, respectively. Therefore, using machine learning to analyze e-nose data is a feasible non-destructive testing method. Ansari [50] focused on using an e-nose with PCA, LDA, SVM, and Naive Bayes Classifier (NBC) as the classifiers. In the experiment, six indoor air pollutants were measured and analyzed and the machine learning technology for the classifiers was divided into supervised and unsupervised. The experimental results showed that when using data sets, SVM had the best effect on the data classification and could successfully achieve feature conversion. Zhu [51] introduced a machine olfactory system for identifying the storage life of hairy crabs. In the experiment, the stacked denoising auto-encoder algorithm (SdA) was used to extract the features sensed by the e-nose sensor and then SVM was used for the classification. The experimental results showed that SdA and SVM achieved the highest identification accuracy rate of 96.67% so this study could provide suggestions for the identification of the storage life of hairy crabs. Shen [52] used an e-nose to detect odors and study shelf life using a non-destructive testing (NDT) method, and then analyzed the detection of fresh fruit based on the optimized e-nose sensor array using SVM and a reverse neural network model. Spinach was used as the object of this experiment to analyze and predict its freshness and chlorophyll. The experimental results showed that e-nose technology based on odor classification and identification can better guarantee the shelf life of fresh fruit. Kit [53] used an e-nose to extract the unique odor characteristics of herbs and an Artificial Neural Network (ANN) to classify the odor data obtained by the e-nose; the e-nose in the experiment was sensed by five gases. The e-nose system had a good effect on the classification of the herbal samples. The accuracy of the classification increased with the number of sensors used. The experimental results showed that as the number of sensors increased, the e-nose significantly improved its sensitivity and accuracy in the herbal classification. Ayari [54] studied the aroma fingerprint of beef ghee and used eight MOS sensors to form an e-nose system. He collected data on pure beef ghee and its mixtures when mixed with five different amounts of margarine and used the PCA and ANN methods for the data analysis and testing. The experimental results showed that the PCA analysis and identification results of the pure beef ghee and mixtures were 84% and 14%, respectively. The accuracy rate achieved using ANN to analyze the samples was 85.6%. When the samples were divided into two groups, the best accuracy was 97.2%. Qiu [5] used an e-nose to collect data on five different processing methods of strawberry juice and used PCA, LDA, Multiple Linear Regression (MSR), PLSR, ELM, and LIBSVM (Library Support Vector Machines) for the classification and regression prediction. The experimental results showed that ELM had a fast learning speed and achieved better performance in the classification and regression tasks. Therefore, combining an e-nose system with an ELM learning model could produce good results. Xu [55] used an e-nose and Computer Vision System (CVS) to detect the quality of tea aroma and appearance. In the experiment, the signals obtained by the e-nose and CVS were fused and processed and then the K-NN, SVM, and multinomial logistic regression (MLR) models were used for the classification. The experimental results showed that K-NN and SVM could be used as classification models. The performance of SVM was better than K-NN. Rasekh [56] used an e-nose with nine MOS sensors to detect and analyze juice and industrial juice and used ANN to analyze and classify the data signals collected by the e-nose sensor. This method could identify natural juices and industrial juices with an accuracy rate of 95%.

To sum up, in recent years, with continuous advancements in artificial olfactory sensor technology and applications, electronic nose technology has made significant progress in its quality assessment and aroma identification of coffee, beverages, and food. However, little attention has been paid to developing electronic nose technology and efficient coffee aroma feature extraction methods to achieve traceability. Therefore, in this work, we developed a method to generate a digital aroma fingerprint for each type of tested coffee bean, which can be used as a biochemical marker of the aroma characteristics.

## 3. System Framework

The e-nose system uses olfactory gas sensors to mimic human olfactory cells as the basic unit of olfactory gas sensing. The major concept in e-nose system design is that each sensor in the olfactory sensor array has a different sensitivity to the same sample under test, which can make different gases produce different patterns in the detection of the olfactory sensor array. The system framework and configuration of the e-nose system we developed, which are consistent with the underlying principles of the electronic nose system, are described in detail below.

### 3.1. System Framework and Workflow

**Figure 1:**
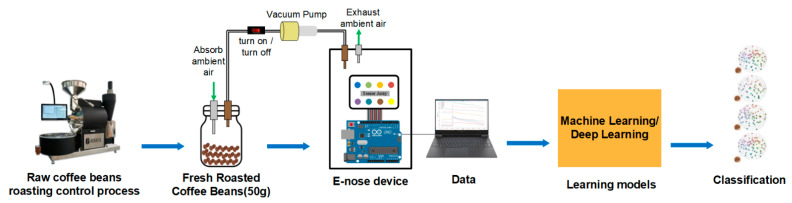
System framework.

Light to light-medium roasts can bring out the original characteristics of coffee. Therefore, for better differentiation, the coffees tested in this did should not exceed light-medium roasts. The system framework diagram is shown in Figure 1. The work began with roasting green coffee beans using consistent roasting conditions, from unflavored green beans to those with a particular aroma. After an appropriate duration of the discharge process, we then took some samples of freshly roasted coffee beans and stored them in the device’s concentrate chamber to collect the resulting coffee aroma. The aromatic gas was then delivered to the e-nose device and interacted with sensors in the e-nose system. Then, the sensor output data were collected and preprocessed to use the algorithm on as much useful data as possible. The extracted aroma dataset was used to train the selected machine learning models for the recognition task. The machine learning model, similar to how the human brain functions, learned the major features of the tested coffee aroma in order to perform the recognition of the coffee aromas. The detailed experimental process is described later.

### 3.2. Experimental Processes

**Figure 2:**
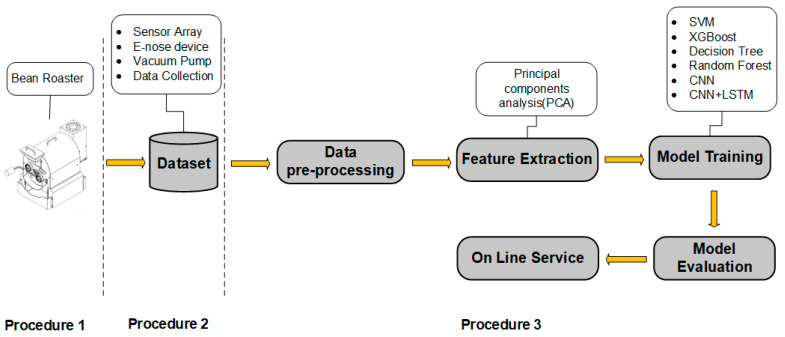
Research workflow and experimental process.

The experimental process was divided into three stages (see Figure 2). The first stage was the process from the coffee roasting to the odor samples collection. Second, some measurements were taken of the roasted coffee beans. During this process, we took some samples of freshly roasted coffee (approximately 50 g) and stored them in the concentration chamber of the device to perform the measurement process. We then tested with ground coffee, during which we took some samples and roasted green coffee beans in a dedicated machine in the lab; once the coffee was properly roasted, it was placed into a coffee grinder and ground to a medium grind. In the third phase of the experiment for the coffee cup test, ground coffee was added to hot water, which helped the measurement process since liquid coffee produces more volatiles. The third stage then involved performing the preprocessing of the odor sample data, feature engineering, model training, and the evaluation of results. The entire experimental process is shown in Figure 2.

### 3.3. Coffee Aroma and E-Nose Sensor Array

The aroma and taste of coffee are mainly produced after high-temperature roasting. During the roasting process, the moisture of the green coffee beans is slowly released and the weight is reduced. Meanwhile, the color deepens, the volume expands, and the aroma-containing oil is slowly released. The large amount of chlorogenic acid originally contained in the green coffee beans gradually disappears during the high-temperature roasting process, releasing delicious fruity acids, and its taste varies according to the duration of roasting. 

Coffee bean-producing areas have a unique smell. Different olfactory gas sensors form an array configuration. When odor molecules of different compositions encounter the olfactory sensor, each sensor has varying degrees of resistance. Different odors and intensities also cause the sensing array to generate different resistance data. We collected the front-end data of each single coffee odor and observed that the olfactory gas sensor corresponded to different coffee odors. The smell receptors of the system are activated when exposed to a coffee odor sample in the system’s sensors. Our e-nose deciphered the activation patterns of these receptors when exposed to the samples.

### 3.4. Configuration of the E-Nose System

**Figure 3:**
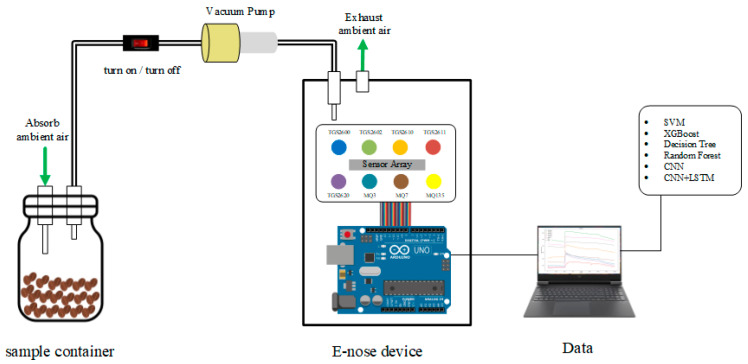
Developed e-nose system prototype and configuration.

In this work, our e-nose system employed a sensor array with the advantages of small size and fast response but also low power consumption and low costs. The sensors sensed volatile organic compounds and smells with an easy divergence under normal temperatures and pressures. The metal–semiconductor oxide material was used as the component and the measurements were carried out by reacting with the gas. The e-nose system contained eight olfactory gas sensors. The system function was connected to the data collector (i.e., Arduino microcontroller board) through the olfactory sensor array. The acquisition of samples included collecting all sensor data every second. After the sample data set was collected, data pre-processing, feature extraction, model training, parameter adjustment, evaluation, and analysis were performed. The gas was captured by the sensor array to be processed by the Arduino board into a digital signal. The e-nose system is shown in Figure 3.

### 3.5. Humidity Effect on the Output Results

Humidity affected the output of our developed e-nose system in several ways, as discussed below.

First, as mentioned earlier, our system framework began with the process of roasting green coffee beans so the output was naturally affected by environmental factors including humidity. In previous studies, it was clear that the humidity and temperature of the environment affected the moisture content of the coffee beans and the gas sensor response. Rao [57] highlighted that green coffee beans needed to be dried to lower the moisture content by between 10.5 and 11.5% before the roasting process. Furthermore, maintaining the relative humidity of the environment in the range of 45–50% and the temperature of between 20 and 22 °C provided the ideal circumstances for producing coffee beans of a higher quality. In addition, during the process of roasting, increasing the speed and humidity of the air changed the flavor of the coffee beans [58].

Second, on the sensor side, when the humidity and temperature in the environment increased, so did the mobility of the water molecules. This made it easier for water molecules to bind to the sensing area of the sensor. As a result, the adsorption capacity was decreased by the thin metal oxide layer on the target gas and sensor surface [59]. Decreasing the sensitivity of the gas sensor ultimately affected the output changes detected by our e-nose system.

Third, in the aspect of the aroma recognition of an e-nose, Brudzewski [30] mentioned that the most important challenge to the efficient performance of an electronic nose used for recognition in the forgery of coffee brands was its sensitivity to small changes in odor, particularly if the measurements are associated with the interference effects occurring due to the changes in humidity or temperature of the environment. It is quite challenging to accurately identify even the slightest variation in odor concentration.

Therefore, in this study, our system for performing coffee aroma recognition experiments was controlled at an appropriate humidity to maintain the accuracy of the output results.

### 3.6. Sensor Selection and Data Extraction

Although more than 850 volatile compounds have been identified in roasted and ground coffee [60], only about 30 of them can be perceived as being related to the flavor and aroma. The aroma impact compounds in roasted coffee powder include Methanethiol, 2-Methylpropanal, 2-Methylbutanal, 2,3-Butanedione, 2,3-Pentanedione, 3-Methyl-2-buten-1-thiol, and 2-Methyl-3-furanthiol, etc. [60]. In addition to the electronic nose method for identifying coffee aroma discussed in this paper, GC–MS (Gas Chromatography–Mass Spectrometry)-based methods are often used for the analysis of volatile aroma compounds in coffee. However, although the instrumental technique using GC–MS is effective for assessing aroma in coffee, the main disadvantage of the GC–MS method is due to expensive equipment and time-consuming experiments. In contrast, electronic nose technology is easier to use and less expensive. Thus, in this study, we did not use GC–MS methods for identifying the volatile compounds in the coffee samples.

#### The Commercial Sensors Chosen for the Developed E-Nose System

Gas sensors are widely used to form sensor arrays for odor measurement. Gas sensor operation involves interactions between gas molecules and sensor coating materials that modulate the current through the sensor, which the sensor then detects and converts the modulation into a recordable electronic signal [61]. For example, in the type of gas sensors used in our system, the gas was sensed by its effect on the resistance of the tin dioxide semiconductor, which was a change in conductivity due to a combustion reaction with the lattice oxygen of the tin dioxide particles [62]. To improve the measured content provided by the sensor array, it was necessary to increase the selectivity (i.e., increase the number of sensors delivering useful new information) to enhance the capabilities of the electronic nose. As a result, considering the measured contents, the sensors selected for composing our e-nose system included TGS2600, TGS2602, TGS2610, TGS2611, TGS2620, MQ135, MQ3, and MQ7. The sensors of series TGS26xx were applied because this family is known for its high stability of operation and small size. The major sensitivities of the measured gas contents for the MQ135, MQ3, and MQ7 sensors were carbon dioxide, ethanol, and carbon monoxide (CO), respectively. The sensors of the developed e-nose system and measured gas contents are shown in Table 1. These types of sensors were chosen because of their high sensitivity to gases including pollutants, carbon dioxide, ethanol, carbon monoxide (CO), etc. (see Table 1).

**Figure 4:**
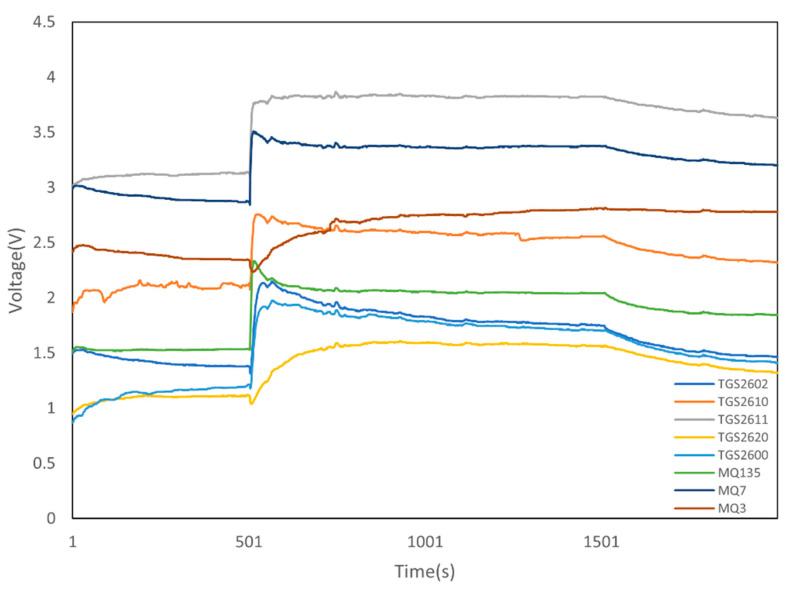
An example of data extraction from our e-nose (8 sensors) outputs.

As shown in Figure 4, our measurement process included the calibration time (500 s before measurement and 1500 s after measurement). Therefore, data extraction was performed to identify stable e-nose signals in each coffee aroma measurement. In Figure 4, from the detected initial values and endpoints (i.e., 500–1500 s), eight subdivisions of the e-nose data were automatically performed to extract the average value of each sensor, which was used as the input for the machine learning modeling.

## 4. Feature Extraction of Aroma Data for Digital Fingerprint Generation

In addition to ensuring the accuracy of the aroma identification, achieving the traceability of the coffee’s origin is also critical. In this work, we aimed to identify the origin of coffee using an e-nose system and machine learning modeling. Therefore, the learning model was developed using the collected sensor data as input to verify the type (origin) of the coffee based on what was reported on the label. As a possible solution to the double-check function of coffee bean origin traceability, we developed a method to establish a digital fingerprint for each tested coffee bean, which could be used to represent its aroma characteristics and be permanently stored in a database. The visual fingerprints extracted from the coffee aromas provided unique traces for identifying their origins.

### Construction of Digital Fingerprints of Measured Coffee Aromas

**Figure 5:**
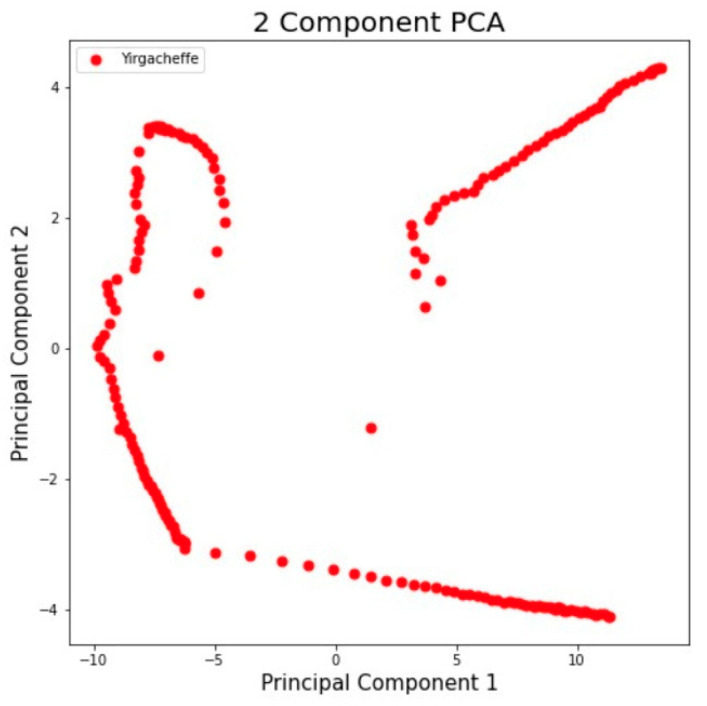
A sample digital fingerprint of coffee aroma.

By capturing the e-nose signals and sending them to the computer, the signals are processed into patterns for specific gases, which are used as the basis of the calculations to identify and confirm the different aromas. Using a suction device to vacuum the odor collector, we took 50 g of freshly roasted coffee beans from the sample. After an appropriate time of exhaust, the data collector passed through the gas sensors TGS2600, TGS2602, TGS2610, TGS2611, TGS2620, MQ135, MQ3, and MQ7 to sample the data in sequence. The measurement time of each sample was 2000 s and the sample was measured again in 2000 s intervals. In this work, we mainly utilized the Principal Component Analysis (PCA) method for the feature extraction. As an efficient dimensionality reduction method, PCA projects the original variables onto new coordinates to obtain the values of a set of linearly uncorrelated variables called “principal components” (PCs), thereby eliminating the overlapping parts of the existing information. Thus, multivariate statistics were employed to discriminate and identify the origins of the coffee samples by performing Z-score normalization and PCA techniques. The original response data of the olfactory sensor were converted into 128 × 128 RGB images in units of 100 data sets. A sample digital fingerprint of coffee aroma is shown in Figure 5.

## 5. Experiments and Results

**Figure 6:**
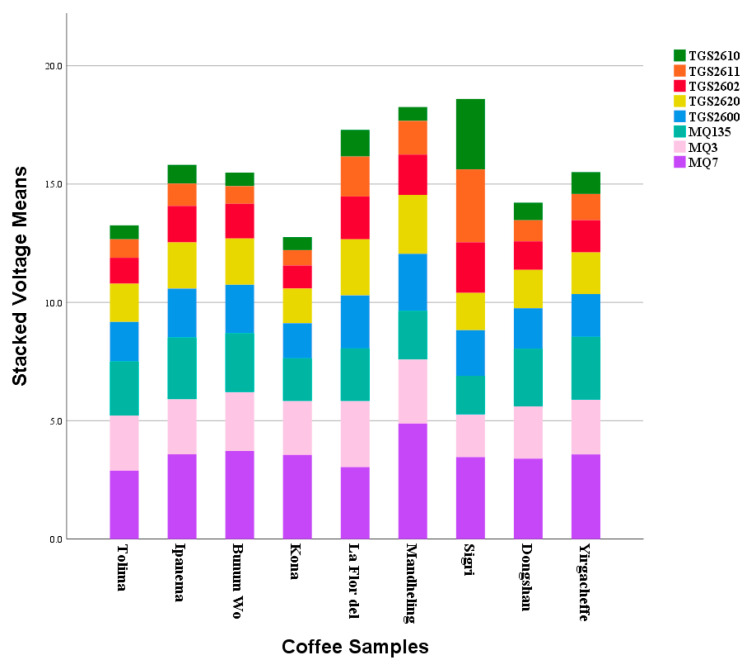
Stacked mean values of the e-nose sensors. Illustration of differences between each sensor for the coffee samples (bar colors).

According to the system framework described previously, the system model experiments with the extracted feature data and several machine learning methods. Figure 6 shows the stacked averages of the electronic nose sensors, highlighting the differences (bar color) between each sensor for the coffee samples calculated by averaging the values for each sensor in each coffee data set using the SPSS software package. It can be observed that the sensor with the highest voltage for all samples is the MQ7 (carbon monoxide (CO)) sensor for Mandheling. The carbon monoxide released during the roasting of coffee makes the MQ7 a suitable sensor. Figure 6 shows that the MQ7 sensor in each coffee sample had the highest voltage.

### 5.1. Experiment 1: Coffee Bean Aroma Recognition Using E-Nose with Manual Gas Extraction

In the first-generation system experiments, we performed a manual extraction of gas to obtain the odor data. Before performing the aroma recognition experiments, the data of the coffee samples were divided into training data (80%) and test data (20%). Thus, in this experiment, for each coffee category, we used 144 samples as training data and 36 samples as test data, adding up to a total of 180 samples. A similar approach was used in previous studies. Given that the dataset was small enough to avoid having enough power to overfit the model, the number of samples used for the model was sufficient.

In this experiment, we employed six machine learning techniques (Decision Tree, Random Forest, XGBoost, SVM, CNN, and CNN+LSTM) to perform the category predictions of the coffee aromas. For each coffee category, two classes of predictions were used to compare two types of coffee (e.g., Kona and non-Kona coffee beans). Nine types of coffee beans, including La Flor del, Kona, Mandheling, Sigri, Bunum Wo, Ipanema, Yirgacheffe, Dongshan, and Tolima were used in this experiment. The classification results were measured in terms of the accuracy achieved by each machine learning method. The accuracy value indicates how accurately the system performed its classifications. The accuracy value was obtained by comparing the correct classification results with the overall data. In Table 2, the accuracy using the selected machine learning algorithms is demonstrated for comparison. The accuracy results ranged from 65.7% to 99.8%, as shown in Table 2.

Then, the experiment using nine-class predictions was conducted by combining all classes into one for the classification. In the nine-class prediction experiment, the accuracy results ranged from 66% to 97%, as shown in Table 3. Thus, the accuracy of the nine-class prediction experiment was slightly lower compared to the two-class prediction experiment (Table 2). This proves that the use of more prediction classes increases the level of difficulty for the classification system.

### 5.2. Experiment 2: Coffee Aroma Recognition Using E-Nose with A Vacuum Pump

To evaluate the proposed coffee aroma recognition system, we employed an e-nose approach with different system configurations. Thus, in the second-generation system experiments, we used a vacuum pump to automatically collect coffee odors instead of manually pumping the gas as in the first-generation experiments. We used the BMP280 pressure sensor to detect the pressure changes in experiments 1 and 2. The changes in pressure affected how much concentration we pumped into the container. The vacuum pump could clearly extract the coffee smell more stably. However, the manual extraction of coffee gas could affect the amount of coffee odor obtained due to the different force and extraction rates, resulting in very obvious pressure changes. To verify the performance improvements, aroma recognition experiments were performed using an e-nose system with a vacuum pump. In this experiment, for each coffee category, the data for the coffee samples were divided into training data (80%) and test data (20%). We used 320 samples as the training data and 80 samples as the test data, for a total of 180 samples. The experimental results are shown in Table 4 and Table 5.

Table 4 lists the experimental results of the two-class predictions for the tested coffee aromas. The accuracy results ranged from 82.9% to 99.8%. Subsequently, the experiment using six-class predictions was carried out by combining all classes into one for classification. In the six-class prediction experiment, the accuracy results ranged from 63% to 96%, as shown in Table 5. Similarly, the accuracy of the six-class prediction experiment was lower than the two-class prediction experiment (Table 4). The use of more classes increased the level of difficulty for the classification system.

### 5.3. Digitizing Coffee Aroma with Proposed Fingerprint Generation Technique

**Figure 7:**
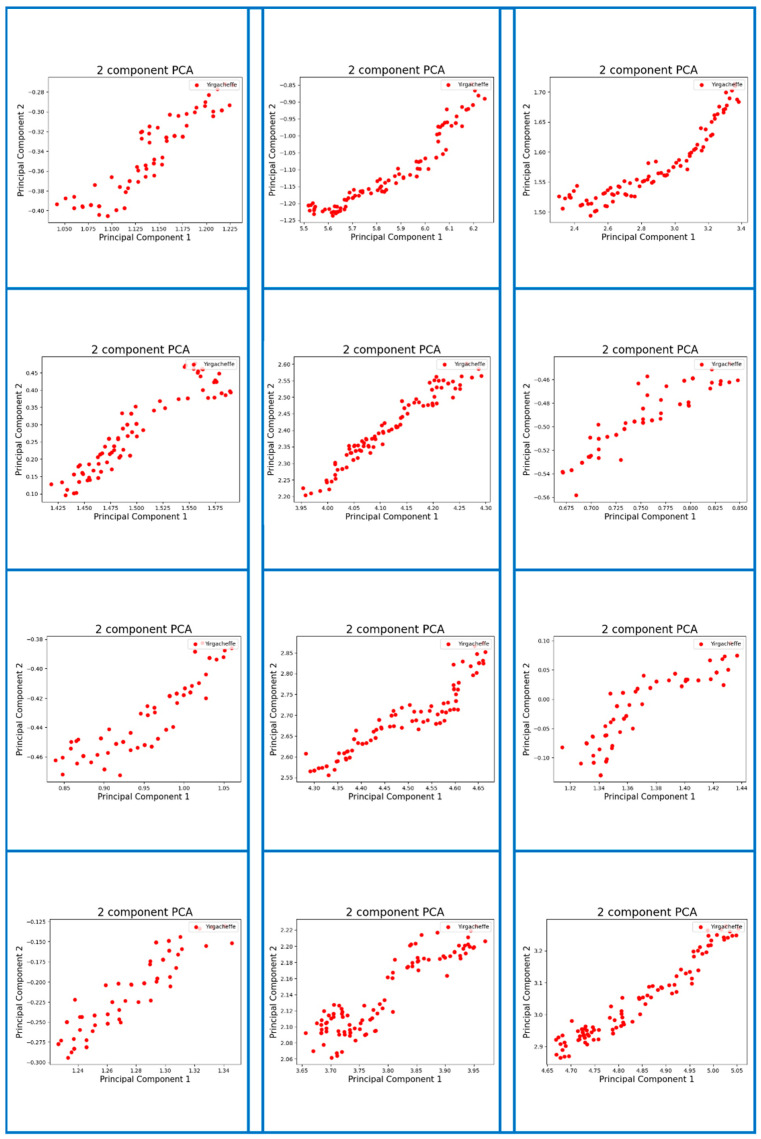
Sample digital fingerprints of coffee aromas originating from Yirgacheffe, Ethiopia.

**Figure A1:**
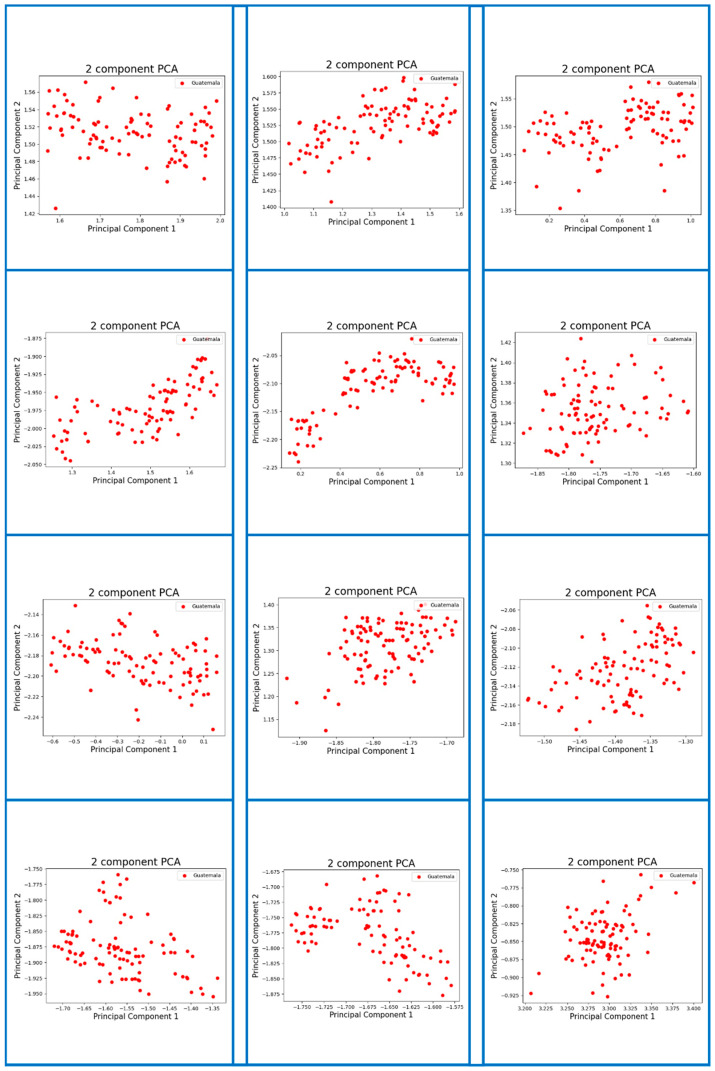
Sample digital fingerprints of coffee aromas originating from Guji Uraga, Ethiopia.

**Figure A2:**
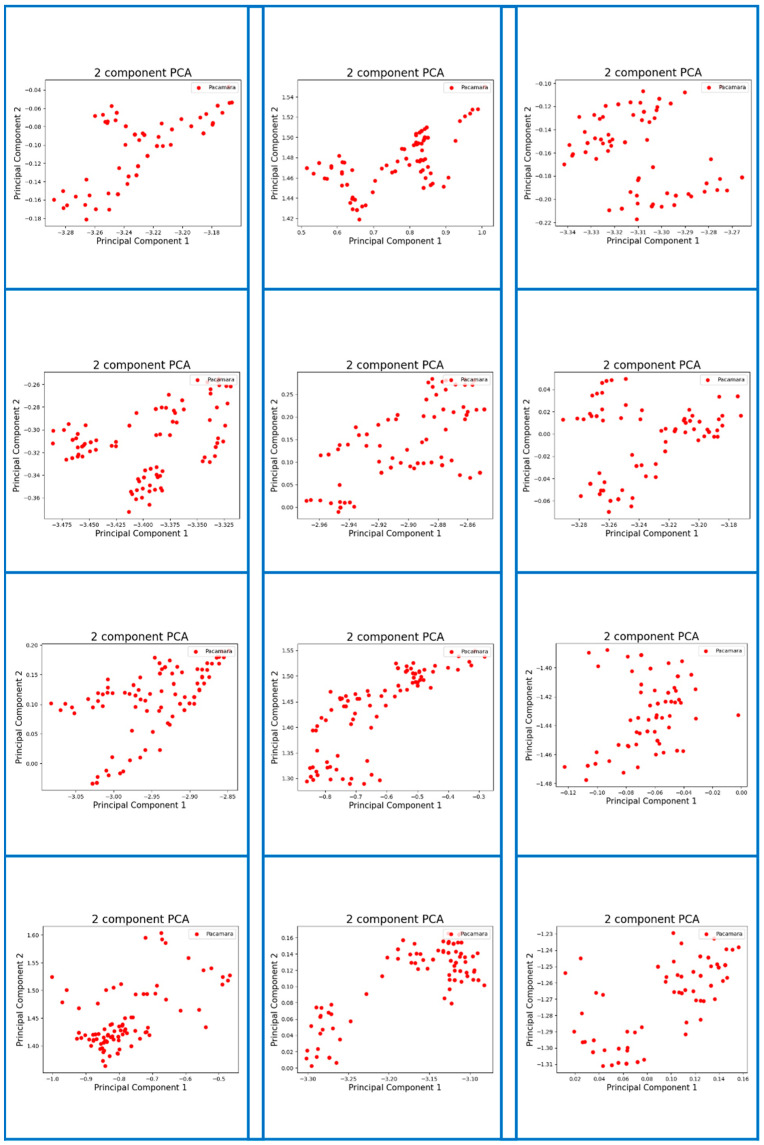
Sample digital fingerprints of coffee aromas originating from Pacamara, Nicaragua.

**Figure A3:**
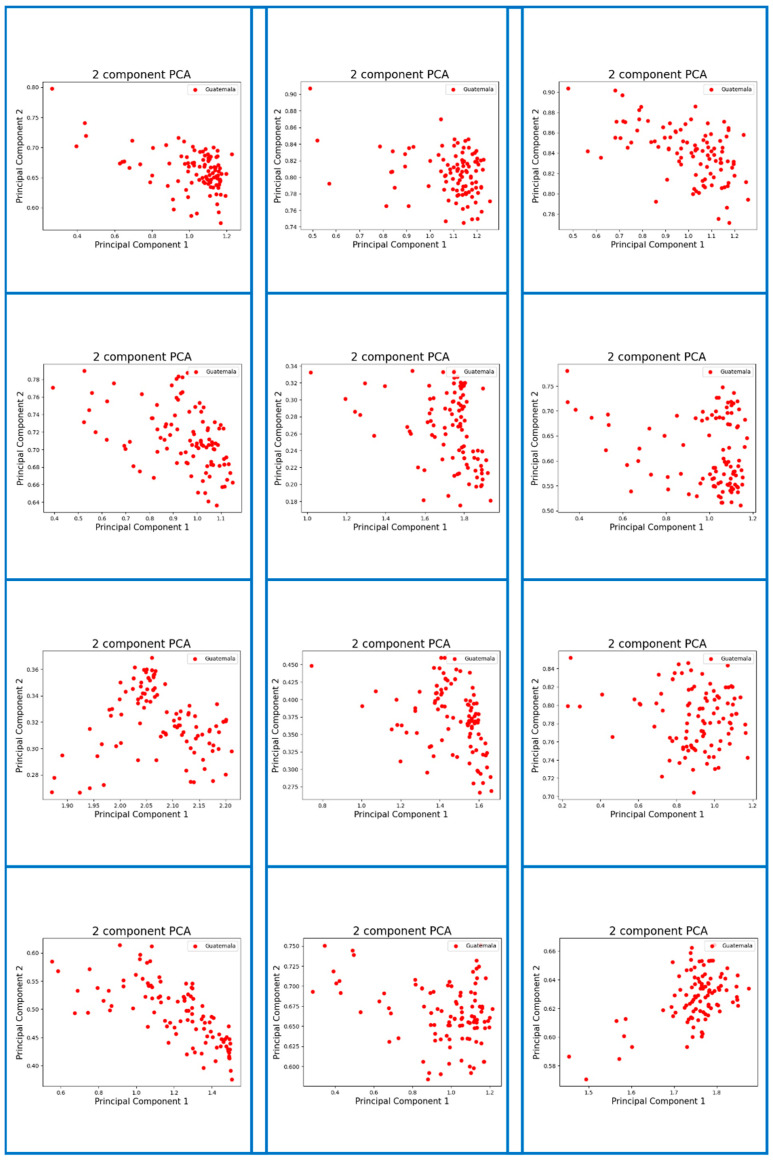
Sample digital fingerprints of coffee aromas originating from Huehuetenango, Guatemala.

**Figure A4:**
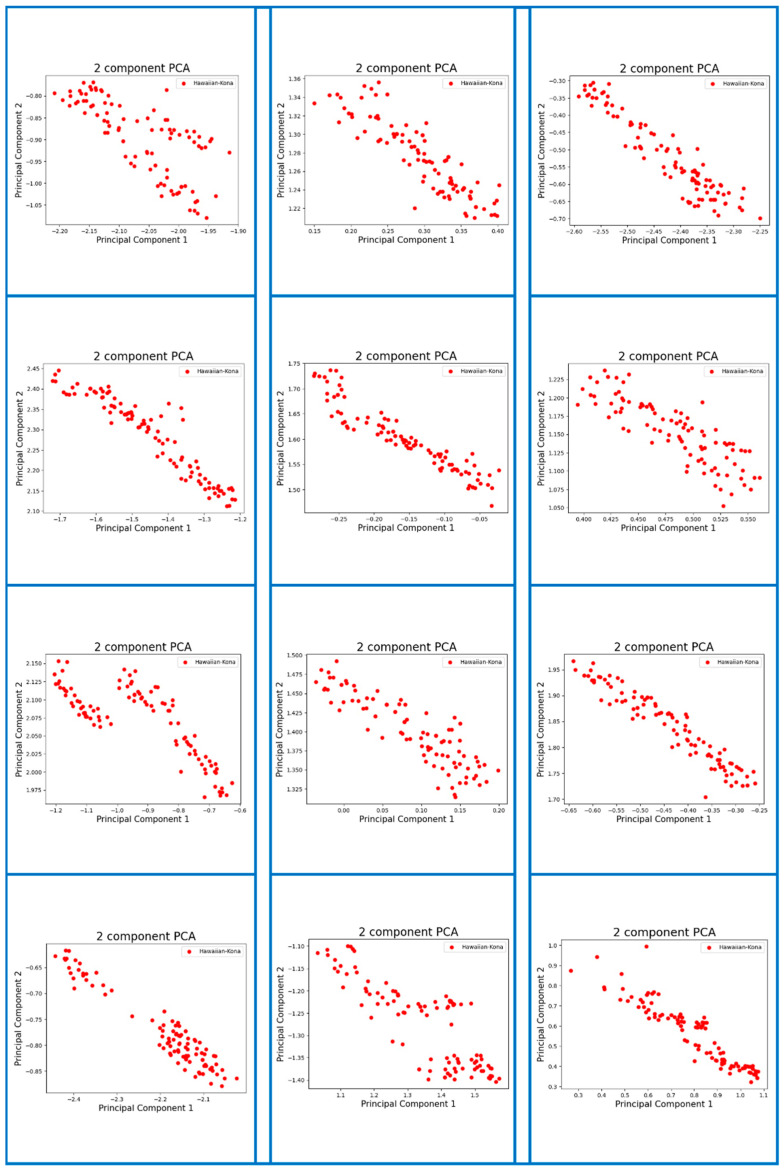
Sample digital fingerprints of coffee aromas originating from Kona, Hawaii.

**Figure A5:**
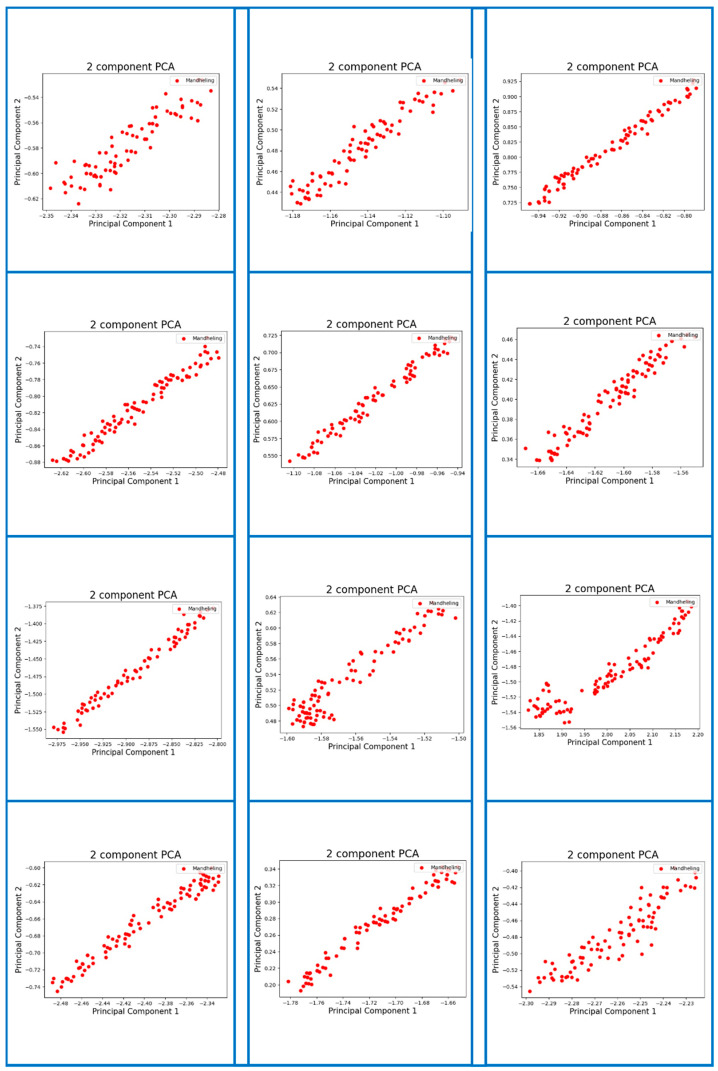
Sample digital fingerprints of coffee aromas originating from Mandheling, Indonesia.

As mentioned previously, the accuracy of the aroma recognition was not the ultimate goal of this work. We also wanted to develop a method to enable the traceability of coffee origin and allow the quality of coffee to be assessed by identifying its aroma features. As a result, we developed a method to generate digital aroma fingerprints for each tested coffee bean, which can be used as the biochemical markers of the aroma signatures. The data extraction method was described in Section 4. Figure 7, Figure A1, Figure A2, Figure A3, Figure A4 and Figure A5 (see Appendix A) show the resulting digital fingerprints of the coffee bean samples.

### 5.4. Discussion

In this work, we combined techniques from olfactory sensor arrays and machine learning techniques to identify the aroma of freshly roasted single-origin coffees from different origins. The experimental results are discussed in detail below.

(1) The experimental results shown in Table 2, Table 3, Table 4 and Table 5 demonstrate the system’s performance in terms of aroma recognition accuracy. The resulting accuracies of the utilized machine learning approaches including Decision Tree, XGBoost, Random Forest, SVM, CNN, and CNN+LSTM are listed for performance comparison. The results are encouraging because they show that the use of machine learning has positive effects and achieves one of the goals of this work. The highest accuracy rate in the tested coffee aromas is 99.8% (see Table 2). This means that our e-nose system can effectively identify coffee aromas.

(2) It is worth mentioning that most existing e-nose applications have a common condition, that is, the e-nose is treated as a black box and has no sense of the chemical processes going on. Thus, experimental validation relies heavily on the sample sets. In reality, it is very important to be aware of the fact that a person could sometimes have a limited sample set and as a result, the initial results could be misleading [63]. However, e-noses are far less expensive compared to classical analytical systems such as GC–MS or the running costs associated with human sensory panels. In particular, our method allows us to cumulatively extract coffee aroma fingerprints in order to build a scalable database containing a wide variety of coffee bean aromas.

(3) As mentioned earlier, the goal of this research work is to develop a method to create individual coffee aroma signatures (i.e., digital fingerprints) to identify their provenance. Meanwhile, the developed aroma fingerprint features of coffee beans could be used to ensure the authenticity of the stated origin of coffee beans using machine learning techniques. The resulting digital fingerprints of the tested coffee bean samples were shown in Figure 7, Figure A1, Figure A2, Figure A3, Figure A4 and Figure A5 (see Appendix A). During coffee production, our method allows the use of the aroma profiles of specific coffee beans, which could maintain provenance as a quality assurance method.

(4) In order to prevent unscrupulous merchants from mislabeling inferior coffee beans as high-priced beans to deceive consumers, the digital fingerprints extracted from the tested coffee aromas can be used as the features of coffee smells to re-identify the beans. Alternatively, these digital fingerprints can be incorporated into the biography (i.e., the production history) of the coffee bean products to enable traceability and reconfirm their origins. At the same time, these fingerprints could be stored in an aroma database, which is a comprehensive library of the aroma characteristics of specific coffee beans. The more aroma fingerprints are collected, the more accurate the identification of various coffee aromas.

(5) Still, we should take a closer look at the technical limitations of electronic noses. In recent years, much work has been done to understand the principles of odorant receptors and the organization of the olfactory system. There is only one odorant receptor on each olfactory receptor cell and it can detect a limited number of substances. For complex odorants composed of multiple odorant molecules, several receptors are activated. The resulting receptor patterns determine our impressions of smells [63]. Therefore, we should have a more correct understanding of the function of the coffee e-nose; it is an attempt to imitate the human sense of smell and compared with its biological inspiration, it provides an alternative view of the concept of coffee aroma volatiles.

## 6. Conclusions

Integrating AI-powered e-nose and digital fingerprint generation methods are shown to be appropriate techniques for digitizing coffee aromas and identifying the authenticity of the origins of coffee beans. From a machine learning perspective, the results are promising, with e-nose systems showing high value in terms of sensing accuracy and the aroma fingerprinting features of coffee beans that can be used to ensure the authenticity of their origins. This means that the method enables us to build a scalable database containing a wide range of coffee aromas by storing the extracted coffee aroma data, thereby improving the recognition accuracy of the system. The more coffee varieties and samples collected, the better the identification accuracy of the system. In this study, our contributions are summarized as follows. We developed an e-nose system to successfully discriminate the aroma of freshly roasted coffee in different production regions. In the experiments, we employed the e-nose system to experiment with various machine learning models on several collected coffee beans such as coffee from Yirgacheffe and Kona. The performance results were good in terms of recognition accuracy. Second, we developed a method to create individual coffee aroma signatures (i.e., digital fingerprints) to identify their provenance. For our future work, in order to increase customer trust, the traceability of coffee will be combined with the digital fingerprints and blockchain technology services that have been developed. This is to ensure that the profile data of the coffee supply chain is authentic and to enhance the quality assurance of the coffee. Thus, customers can be assured of the origins of their coffee. This approach can help to ensure that farmers are paid fairly for their crops and maintain sustainable practices. On the other hand, combining a coffee aroma data cloud with edge computing is another promising approach to processing the collected coffee aroma data for real-time aroma discrimination.

## Figures and Tables

**Table 1:** Sensors of the developed e-nose system.

Sensor Name	Measured Gas Contents
TGS2600	Air pollutants (hydrogen, ethanol)
TGS2602	Air pollutants (VOCs, ammonia, H2S)
TGS2610	Butane, Propane
TGS2611	Methane
TGS2620	Alcohol, Solvent vapors
MQ135	Ammonia, Sulfide, Benzene
MQ3	Alcohol, Ethanol
MQ7	Carbon monoxide (CO)

**Table 2:** Classifier performance results in scenario using 2-class predictions (experiment 1).

Origin (Growing Area)	Samples	Decision Tree	Random Forest	XGBoost	SVM	CNN	CNN + LSTM
		Accuracy	Accuracy	Accuracy	Accuracy	Accuracy	Accuracy
**La Flor del** **(Guatemala)**	Training: 144 Test: 36	0.997	0.988	**0.998**	0.983	0.758	0.821
**Kona** **(Hawaiian)**	Training: 144 Test: 36	0.971	0.978	0.969	0.934	0.747	0.80
**Mandhelin** **g** **(Indonesia)**	Training: 144 Test: 36	0.993	0.984	0.995	0.986	0.942	0.989
**Sigri** **(Papua New Guinea)**	Training: 144 Test: 36	0.989	0.973	0.975	0.964	0.885	0.844
**Bunum W** **o** **(Papua New Guinea)**	Training: 144 Test: 36	0.969	0.971	0.969	0.953	0.867	0.924
**Ipanem** **a** **(Brazil)**	Training: 144 Test: 36	0.952	0.966	0.944	0.895	0.923	0.971
**Yirgacheff** **e** **(Ethiopia)**	Training: 144 Test: 36	0.951	0.971	0.941	0.886	0.954	0.983
**Dongsha** **n** **(Taiwan)**	Training: 144 Test: 36	0.942	0.964	0.944	0.889	0.867	**0.657**
**Tolim** **a** **(Colombia)**	Training: 144 Test: 36	0.989	0.991	0.985	0.950	0.987	0.835

**Table 3:** Classifier performance results in scenario using 9-class predictions (experiment 1).

Origin (Growing Area)	Samples	Decision Tree	Random Forest	XGBoost	SVM	CNN	CNN + LSTM
		Accuracy	Accuracy	Accuracy	Accuracy	Accuracy	Accuracy
**La Flor del** **(Guatemala)**	Training:144 Test:36	0.86	0.91	0.86	0.86	0.8	0.8
**Kona** **(Hawaiian)**	Training:144 Test:36	0.9	0.83	0.88	0.85	0.95	0.89
**Mandheling** **(Indonesia)**	Training:144 Test:36	0.94	0.81	0.89	0.72	0.67	0.67
**Sigri** **(Papua New Guinea)**	Training: 144 Test: 36	0.79	0.91	0.75	0.91	0.7	0.71
**Bunum Wo** **(Papua New Guinea)**	Training: 144 Test: 36	0.83	0.86	0.88	0.84	0.76	0.76
**Ipanema** **(Brazil)**	Training: 144 Test: 36	0.8	0.87	0.88	0.91	0.93	0.96
**Yirgacheff** **e** **(Ethiopia)**	Training: 144 Test: 36	0.85	0.92	0.73	0.93	**0.66**	0.73
**Dongshan** **(Taiwan)**	Training: 144 Test: 36	0.73	0.95	0.93	0.85	**0.97**	0.86
**Tolima** **(Colombia)**	Training: 144 Test: 36	0.76	0.84	0.79	0.83	0.94	0.94

**Table 4:** Classifier performance results in scenario using 2-class-predictions (experiment 2).

Origin (Growing Area)	Samples	Decision Tree	Random Forest	XGBoost	SVM	CNN	CNN + LSTM
		Accuracy	Accuracy	Accuracy	Accuracy	Accuracy	Accuracy
**PacaMara** **(Nicaragua)**	Training: 320 Test: 80	0.990	0.985	0.988	0.967	0.904	0.889
**Huehuetenango** **(Guatemala)**	Training: 320 Test: 80	0.968	0.995	0.966	0.952	0.897	0.928
**Yirgacheffe** **(Ethiopia)**	Training: 320 Test: 80	0.986	0.982	0.982	0.963	0.997	0.992
**Mandheling** **(Indonesia)**	Training: 320 Test: 80	0.985	0.985	0.986	0.979	0.989	0.972
**Kona** **(Hawaiian)**	Training: 320 Test: 80	0.986	0.992	0.987	0.987	0.985	**0.829**
**Guji Uraga** **(Ethiopia)**	Training: 320 Test: 80	0.992	0.987	0.982	0.986	**0.998**	0.966

**Table 5:** Classifier performance results in scenario using 6-class predictions (experiment 2).

Origin (Growing Area)	Samples	Decision Tree	Random Forest	XGBoost	SVM	CNN	CNN + LSTM
		Accuracy	Accuracy	Accuracy	Accuracy	Accuracy	Accuracy
**PacaMara** **(Nicaragua)**	Training: 320 Test: 80	0.72	0.7	0.71	0.75	0.7	0.7
**Huehuetenango** **(Guatemala)**	Training: 320 Test: 80	0.83	0.88	0.83	0.87	0.92	0.75
**Yirgacheffe** **(Ethiopia)**	Training: 320 Test: 80	0.94	0.84	0.84	0.88	0.74	0.79
**Mandheling** **(Indonesia)**	Training: 320 Test: 80	**0.63**	0.72	0.81	0.73	0.72	0.82
**Kona** **(Hawaiian)**	Training: 320 Test: 80	0.82	0.86	0.8	0.77	0.93	0.71
**Guji Uraga** **(Ethiopia)**	Training: 320 Test: 80	0.79	0.81	0.89	0.91	**0.96**	0.92

## Data Availability

Not applicable.
